# Utilization of tetanus and diphtheria serology tests in Alberta, Canada: Patterns and implications

**DOI:** 10.1371/journal.pone.0336690

**Published:** 2025-11-21

**Authors:** Jamil N. Kanji, Behzad Heibati, Nathan Zelyas, Hong Yuan Zhou, Gregory J. Tyrrell, Adil Adatia

**Affiliations:** 1 Public Health Laboratory, Alberta Precision Laboratories, Calgary, Alberta, Canada; 2 Division of Infectious Diseases, Department of Medicine, Cumming School of Medicine, University of Calgary, Calgary, Alberta, Canada; 3 Department of Pathology and Laboratory Medicine, Cumming School of Medicine, University of Calgary, Calgary, Alberta, Canada; 4 Department of Laboratory Medicine and Pathology, Faculty of Medicine and Dentistry, University of Alberta, Edmonton, Alberta, Canada; 5 Division of Pulmonary Medicine, Department of Medicine, University of Alberta, Edmonton, Alberta, Canada; Shiraz University of Medical Sciences, IRAN, ISLAMIC REPUBLIC OF

## Abstract

**Background:**

Tetanus and diphtheria (Td) antibody titers can be measured to assess for seroprotection from immunization, though this is not routinely indicated. There are limited population level data on the utilization of these tests and their results.

**Methods:**

This is a population level retrospective study based on laboratory data collected from patients who underwent Td antibody testing. Td IgG titer requests from May 1, 2023, to December 31, 2024, were extracted from the provincial health information system of Alberta, Canada. Td anti-toxin test requests, geometric mean titers, and vaccination status of patients who underwent testing were analyzed. Individuals with multiple tests were assessed for changes in antibody levels, and the proportion of tested individuals who were vaccinated within the past 10 years was calculated. Geometric mean titers were interpreted in relation to established thresholds for long-term protective immunity.

**Results:**

A total of 2,550 patients underwent testing for tetanus (n = 2,349) and diphtheria (n = 2,093) anti-toxin antibody levels. Geometric mean titers varied widely across physician specialties, with pediatrics and Immunology showing higher proportions of recent vaccinations and higher geometric mean titers, while general practice and nephrology had lower values. Nearly 40% of diphtheria test orders in patients immunized within the past 10 years were requested by general practice (n = 336). In contrast, less than 20% of tests were ordered by nephrology (n = 153), pediatrics (n = 95), and pharmacy (n = 12).

**Conclusion:**

Over half the tests were requested by general practice. Our study highlighted variability in vaccination patterns and immune responses across specialties. While antibody testing is useful for assessing protection, a considerable number of tests were performed in individuals likely to be protected by recent vaccination, pointing to inefficiencies and unnecessary healthcare spending. These findings underscore the importance of aligning test ordering practices with immunization history to optimize resource use, avoid redundant testing, support diagnostic stewardship, and inform more cost-effective public health strategies.

## Introduction

Tetanus and diphtheria (Td) remain significant public health concerns globally, despite the widespread success of immunization programs in reducing the incidence of these potentially life-threatening infections [1,2]. The assessment of immunity to these pathogens is primarily based on documented vaccination history; however, serologic testing for tetanus and diphtheria anti-toxin antibodies is occasionally performed to evaluate immune status, particularly in individuals with uncertain vaccination records, suspected immunodeficiency [3,4], or in specific occupational groups such as laboratory workers [5,6].

Current national and international guidelines generally advise against routine pre- or post-vaccination serologic testing in healthy individuals, citing the high efficacy and durability of vaccine-induced immunity [7–9]. Protective antibody thresholds are well established, with levels of ≥0.01 IU/mL for both tetanus and diphtheria typically considered seroprotective, though some guidelines use a higher threshold of ≥0.1 IU/mL for complete protection and consider values from 0.01 to 0.1 IU/mL to be partially protective [5,10]. Despite these recommendations, serologic testing continues to be utilized in clinical practice for a variety of indications, and the appropriateness of its use remains a topic of ongoing discussion.

Previous studies have highlighted that the majority of individuals maintain protective antibody levels for years following vaccination [5,10,11], with estimated half-lives of approximately 11 years for tetanus and 19 years for diphtheria. Despite this long-lasting immunity, current guidelines recommend booster doses every 10 years [12]. As a result, routine serologic testing has limited utility in guiding clinical management outside select populations such as the immunocompromised. Nevertheless, the actual patterns of utilization of Td serologic testing in real-world settings, and the extent to which testing aligns with established guidelines, have not been well characterized at the population level.

To address this gap, we conducted a retrospective review of all Td serologic tests performed in the province of Alberta between May 1, 2023, and December 31, 2024. This study aims to describe the frequency, indications, and outcomes of serologic testing, and to assess the appropriateness of test utilization in relation to current best practice recommendations. It also highlights patterns of Td antibody testing and potential overuse in recently vaccinated individuals, providing evidence to support diagnostic stewardship and inform policy aimed at reducing unnecessary healthcare spending.

## Methods

### Setting and anti-toxin IgG testing

Td anti-toxin IgG testing in Alberta, Canada, is conducted at the Public Health Laboratory (ProvLab) (Alberta Precision Laboratories, Edmonton, Alberta) using the Anti-Tetanus Toxoid ELISA (IgG), and the Anti-Diphtheria Toxoid ELISA (IgG) kits (EuroImmun Medizinische Labordiagnostika AG, Lubeck, Germany) on the DSX® Automated System (Neogen, Lansing, MI, USA). These assays determine quantitative anti-toxin IgG titers (S1 Table). Testing for these titers can be requested by physicians, nurse practitioners, and pharmacists without any restrictions.

Alberta was selected as the study setting due to the availability of comprehensive, province-wide laboratory and immunization data through a single-payer health system, which allows for population-level analysis of Td serologic testing practices. The population in Alberta in 2023–2024 was estimated at 4.7 million persons [13]. The provincial ministry of health follows a standard immunization schedule with boosters for Td (usually combined with acellular pertussis) advised every 10 years and one dose during every pregnancy following completion of the primary series (https://www.alberta.ca/immunization-routine-schedule). Td serology testing from a vaccination perspective in Alberta is only advised in the setting of atypical or large local reactions following one of the first three doses in the primary Td series (https://open.alberta.ca/publications/aefi-policy-for-alberta-immunization-providers).

This study was reviewed and approved by the Conjoint Health Research Ethics Board (CHREB, University of Calgary, Calgary, Alberta, Canada), study ID REB25-0409. The CHREB is considered a provincial research ethics board under the Provincial Harmonization Agreement. The data were accessed for research purposes on May 7, 2025. The authors did not have access to information that could identify individual participants during or after data collection. All data were fully de-identified before being provided for analysis, in compliance with institutional and ethical guidelines.

### Data extraction and chart review

A line-listing of all Td anti-toxin IgG requests from May 1, 2023-December 31, 2024, was extracted from the Alberta Health Services information system. This system includes the ProvLab laboratory information system, which uses Epic EMR from Epic Systems in Verona, WI, USA. May 1, 2023, was chosen as the start of the extraction because it coincided with the go-live date of ProvLab using this HIS. We extracted basic demographics (date of birth, gender as recorded in the HIS, date of test request, date of receipt, postal code of residence, name of ordering clinician, and testing result). The specialty of the ordering clinician was determined using a combination of the HIS and the website of the College of Physicians and Surgeons of Alberta (https://cpsa.ca/). The receipt dates of any tetanus/diphtheria-toxoid containing vaccines were manually extracted from the HIS by one reviewer (JK) for each individual tested.

In Alberta, only vaccine doses given within the province are recorded in this system. Patients can submit previous vaccine records from elsewhere for review, validation and data entry (https://myhealth.alberta.ca/Topic/Immunization/pages/record-submission.aspx).

### Data analysis

Descriptive statistics were calculated to summarize patient demographics. To assess repeated measurements, we grouped data by patient ID and component type (i.e., tetanus and diphtheria), identifying patients with multiple test results. To assess whether Td titer measurement could have been avoided by reviewing vaccine records, the proportion of tests requested in those with confirmed Td vaccinations in the past 10 years were calculated and stratified by order practitioner type (excluding immunology since assessment of Td titers in immunized individuals is a key component of standard humoral immunity assessment) [11]. Td titers results were categorized as non-protective, partially protective, and completely protective using the thresholds <0.01 IU/mL, ≥ 0.01 to <0.1 IU/mL, and ≥0.1 IU/mL, respectively [5,10]. We performed independent two-sample t-tests on the log-transformed antibody concentrations to compare Td antibody titers between age groups and between sexes. All statistical tests were two-sided, and a p-value of <0.05 was considered statistically significant. Antibody titers were summarized using geometric means, as this approach is appropriate for skewed serologic data [14]. All data analyses were conducted using R (version 4.3.2).

## Results

### Testing volumes and patient demographics

A total of 4,442 tests were conducted across 2,550 individual patients. Table 1 shows the type of tests, repeat testing, and patient characteristics such as age and sex. The mean age was 37.5 years (SD = 17.6) for females and 36.9 years (SD = 21.8) for males. Of the 2,550 patients tested, 11.1% had diphtheria anti-toxin IgG testing only, 20.9% had tetanus anti-toxin IgG only, and 67.9% received both tests. Repeat testing during the study period was observed in 3.5% of diphtheria tests (79/2,266) and 3.6% of tetanus tests (73/2,016). There were 1,153 (45.2%) individuals who did not have available immunization records with 947 diphtheria and 984 tetanus anti-toxin tests recorded among them. Among individuals who were tested for antibody titers more than once and received a vaccination between the two tests (n = 4 for diphtheria and n = 2 for tetanus), there was a noticeable increase in geometric mean titers for both diphtheria and tetanus. For diphtheria, the geometric mean titer increased from 0.97 IU/mL (SD = 0.16) at the first test to 1.81 IU/mL (SD = 0.41) at the second test, indicating nearly a two-fold increase. For tetanus, the increase was even more pronounced, with geometric mean titers rising from 0.18 (SD = 0.00) to 3.31 IU/mL (SD = 2.11) (S1 Fig).

**Table 1:** Summary characteristics of patients (n = 2550).

	Variable	Value
**Number of tests conducted**	Total Td tests	4,442
	Diphtheria anti-toxoid IgG	2,093 (47.1%)
	Tetanus anti-toxoid IgG	2,349 (52.9%)
**Patients tested (n = 2,550)**	Diphtheria anti-toxoid IgG only	284 (11.1%)
	Tetanus anti-toxoid IgG only	534 (20.9%)
	Both tests	1,732 (67.9%)
**Repeated tests** ^†^	Diphtheria anti-toxoid IgG	79/2266 (3.5%)
	Tetanus anti-toxoid IgG	73/2016 (3.6%)
**Gender**	Female	1,551 (60.8%)
	Male	998 (39.1%)
	Gender not recorded	1 (0.04%)
**Age (years)**	Median [IQR]	36 [23–50]
	Median age for females (range)	36 (1–96)
	Median age for males (range)	36 (1–85)
	Mean age for females (SD)	37.5 (17.6)
	Mean age for males (SD)	36.9 (21.8)
**Patients with no vaccination history available**		1,153 (45.2%)

†Number of tests performed in patients who had > 1 test recorded in the study period.

SD: Standard Deviation.

### Proportion of tests ordered by each physician specialty

**Fig 1:**
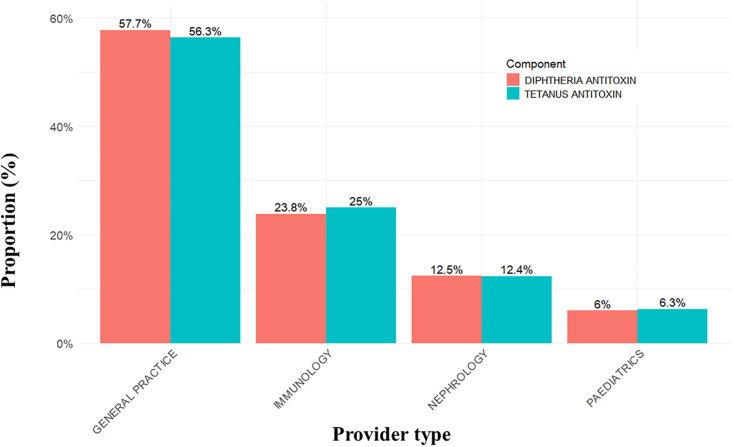
Proportion of diphtheria and tetanus antibody tests stratified by ordering medical specialty.

Fig 1 shows the proportion of Td tests ordered by various physician specialties and allied health providers. Most of the diphtheria (n = 1,163) and tetanus (n = 1,235) tests were requested by general practice, accounting for 57.7% and 56.3% of test requests, respectively. This was followed by immunology (23.8% diphtheria and 25% tetanus test orders), and nephrology (12.5% diphtheria and 12.4% tetanus test orders), respectively. Additional specialties accounting for >2% of tetanus and diphtheria test orders were pediatrics, pharmacy, and hematology. The remaining specialties, including travel medicine, critical care, otolaryngology, obstetrics, medical genetics, and cardiovascular surgery, each contributed less than 5 test requests during the study period (S2 Table).

### Geometric mean of Td titers stratified by ordering physician specialty

**Fig 2:**
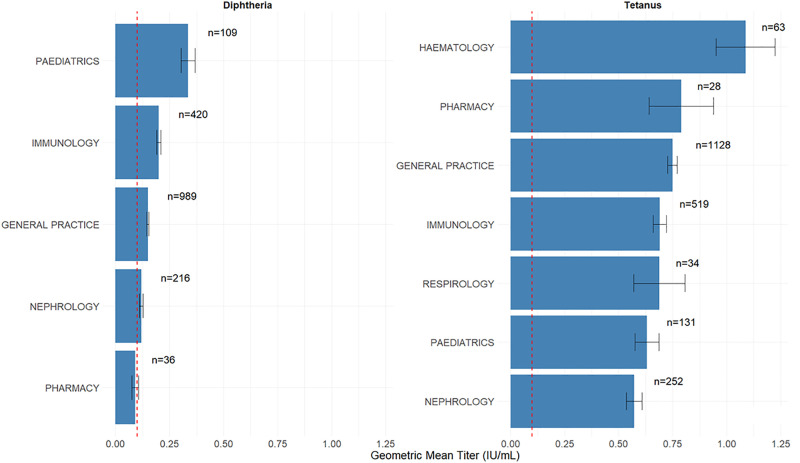
Geometric means of diphtheria and tetanus titers stratified by ordering medical specialty. Error bars represent standard errors. In cases where patients were tested more than once, only the first measurement result was included. The dashed red line indicates the 0.1 IU/mL threshold.

Fig 2 shows the geometric mean of Td antibody titers stratified by physician specialty, based on unique patients (in patients in whom Td antibody titers were measured more than once during the study period, the first result was used).

Among specialties ordering >10 tests, the geometric mean titers were highest for those ordered by pediatrics (0.3 IU/mL), followed by immunology (0.2 IU/mL), general practice (0.15 IU/mL), and nephrology and pharmacy, both below 0.15 IU/mL. For these specialties, the proportion of tests ordered that showed the patient had complete protection (>0.1 IU/mL) was 78.9% for pediatrics, 63.1% for immunology, 54.7% for general practice, 47.2% for nephrology and 27.8% for pharmacy. Using a cutoff of 0.01 IU/mL the proportion of tests results showing protection was 100% for pediatrics, 95.7% for immunology, 94.7% for general practice, 94.9% for nephrology and 97.2% for pharmacy.

For tetanus antibody, among specialties ordering >10 tests, the geometric mean titers for tetanus anti-toxin were highest for those ordered by hematology (1.08 IU/mL), followed by pharmacy (0.79 IU/mL), general practice (0.75 IU/mL), and immunology (0.69 IU/mL). For these specialties, the proportion of tests ordered that showed the patient had complete protection (>0.1 IU/mL) was 92.1% for hematology, 89.3% for pharmacy, 88.4% for general practice, and 90.6% for immunology. Using a cutoff of 0.01 IU/mL the proportion of tests results showing protection was 100% for hematology, 96.4% for pharmacy, 98.2% for general practice, and 99.2% for immunology.

### Geometric mean of Td titer stratified by ordering physician specialty among those who had confirmed records of receiving a vaccine

**Fig 3:**
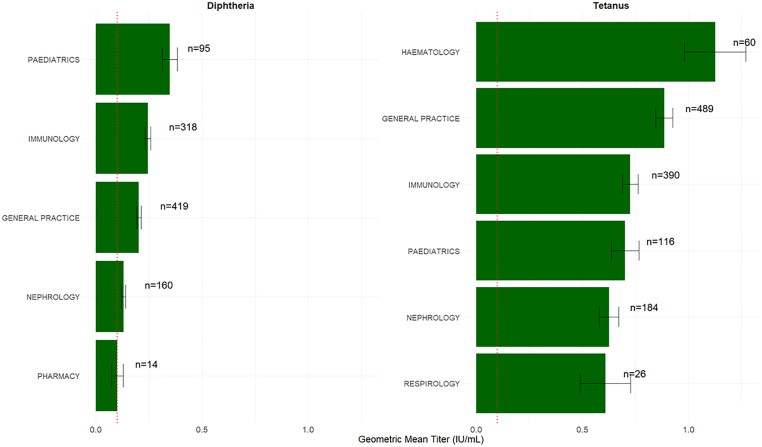
Geometric mean of diphtheria and tetanus antibody titers by ordering medical specialty amongst those with confirmed vaccine records. Error bars represent standard errors. In cases where patients were tested more than once, only the first measurement result was included. The dashed red line indicates the 0.1 IU/mL threshold.

Fig 3 shows the geometric mean Td antibody titers stratified by ordering practitioner specialty for the n = 1396 (54.8%) patients who had available vaccine records confirming receipt of Td immunization.

Among specialties ordering >10 tests, the geometric mean titers for diphtheria were highest for those ordered by pediatrics (0.35 IU/mL), followed by immunology (0.25 IU/mL), general practice (0.20 IU/mL), nephrology (0.13 IU/mL), and pharmacy (0.10 IU/mL). For these specialties, the proportion of tests ordered that showed the patient had complete protection (>0.1 IU/mL) was 80% for pediatrics, 69.8% for immunology, 63.7% for general practice, 50.6% for nephrology and 35.7% for pharmacy. Using a cutoff of 0.01 IU/mL the proportion of tests results showing protection was 100% for pediatrics, 97.5% for immunology, 96.4% for general practice, 95.6% for nephrology and 92.8% for pharmacy.

For tetanus antibody titer, among specialties ordering >10 tests, the geometric mean titers were highest for those ordered by hematology (1.12 IU/mL), followed by general practice (0.88 IU/mL), immunology (0.73 IU/mL), pediatrics (0.70 IU/mL), nephrology (0.62 IU/mL), and respirology (0.61 IU/mL). For these specialties, the proportion of tests ordered that showed the patient had complete protection (>0.1 IU/mL) was 93.3% for hematology, 93.2% for general practice, 91.5% for immunology, 90.5% for pediatrics, 85.3% for nephrology, and 80.8% for respirology. Using a cutoff of 0.01 IU/mL the proportion of tests results showing protection was 100% for hematology, 98.9% for general practice, 99.5% for immunology, 100% for pediatrics, 99.5% for nephrology, and 100% for respirology.

### Proportion of tests ordered for patients immunized in the last 10 years

**Fig 4:**
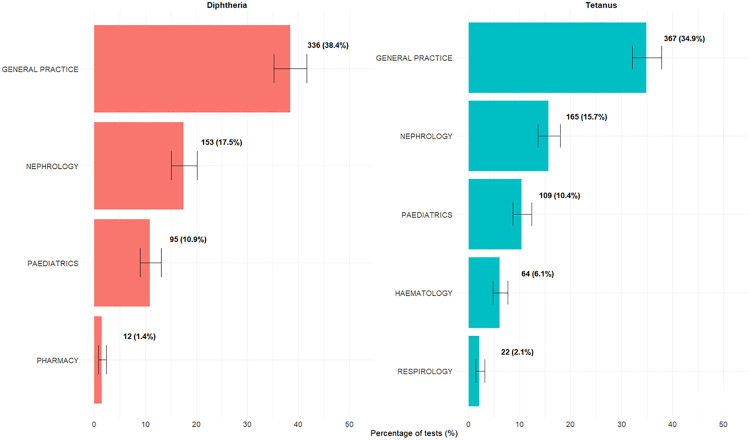
Proportion of tests obtained in patients with confirmed tetanus and diphtheria vaccination within previous 10 years by provider type. Total number of tests for tetanus and diphtheria anti-toxin were 1,051 and 875, respectively, including repeat tests performed in the same patients.

Fig 4 shows the proportion of tests reported in patients who had vaccine records confirming immunization within the past 10 years, stratified by physician specialty. There were 772 diphtheria and 934 tetanus tests associated with confirmed vaccination within the past 10 years. For diphtheria anti-toxin, general practice ordered the highest proportion of tests among patients vaccinated within the past 10 years (38.4%, n = 336). This was followed by nephrology (n = 153), pediatrics (n = 95), and pharmacy (n = 12), all below 20%. Similarly, for tetanus anti-toxin, general practice (34.9%, n = 367) and immunology (29%, n = 305) had the highest rates of tests ordered. Nephrology (n = 165) and pediatrics (n = 109) followed with intermediate proportions tests ordered, while hematology (n = 64) and respirology (n = 22) had lower proportions, with most under 10%.

Among patients tested for diphtheria anti-toxin, those vaccinated within the past 10 years had a higher proportion of protective antibody levels (≥ 0.1 IU/mL) compared to those vaccinated more than 10 years ago (66.6% (510/766) vs. 54.4% (550/1011)). A similar pattern was observed for tetanus anti-toxin, with 91.7% (907/989) of recently vaccinated patients showing protective levels (≥0.1 IU/mL), compared to 88.1% (1063/1206) among those vaccinated more than 10 years ago.

### Impact of age and sex on Td antibodies

**Fig 5:**
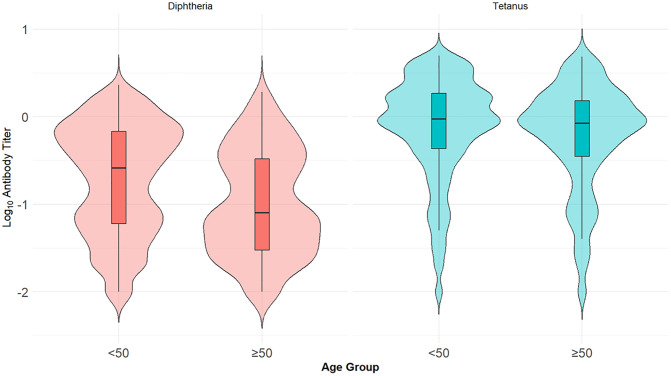
Distribution of tetanus and diphtheria anti-toxin titers by age group (<50 vs ≥ 50 years; Tetanus: n = 1709/ < 50, n = 639/ ≥ 50; Diphtheria: n = 1583/ < 50, n = 509/ ≥ 50).

We compared geometric mean titers of Td antibodies between individuals aged below 50 and those aged 50 and above. The mean diphtheria anti-toxin antibody titer was significantly higher in patients <50 years (0.37 IU/mL ± 0.45) compared to those ≥50 years (0.20 IU/mL ± 0.33) (p < 0.0001). Similarly, tetanus anti-toxin antibody titers were also higher in the younger age group (1.32 IU/mL ± 1.24) than in the older group (1.07 IU/mL ± 1.09) (p < 0.0001) (Fig 5). In addition, we examined whether there were differences in Td antibody titers between male and female patients. For diphtheria, the mean titer was significantly higher in males (0.36 ± 0.46 IU/mL) compared to females (0.31 ± 0.41 IU/mL; *p* = 0.014). In contrast, for tetanus, there was no significant difference in antibody titers between sexes; mean titer was 1.28 ± 1.25 IU/mL in males versus 1.23 ± 1.17 IU/mL in females (*p* = 0.353).

## Discussion

We conducted a population level retrospective study of Td serological tests performed in the province of Alberta, Canada. Our findings suggest that a substantial proportion of diphtheria and tetanus anti-toxin antibody tests were ordered for patients who were immunized with Td within the past 10 years despite existing guidelines that discourage routine titer testing in fully vaccinated, low-risk individuals due to the long-lasting nature of vaccine-induced immunity [15,16]. For example, nearly 40% of Td test orders from general practitioners were for recently vaccinated patients, while specialties such as hematology, nephrology, and pharmacy also showed notable rates of potentially redundant testing.

Measurement of vaccine titers is an established component of the laboratory assessment of primary and secondary antibody deficiencies [4,17–19]. In the case of common variable immunodeficiency, IgG subclass deficiency, and specific antibody deficiency, the demonstration of impaired specific antibody production to vaccine antigens is required for diagnostic confirmation. Td serology is commonly used to assess specific antibody production to T cell-dependent peptide antigens, which likely account for the significant test utilization by immunology and hematology (together accounting for 24.8% of tests). Interestingly, there were only 2 patients that underwent diagnostic vaccination in which Td titers were measured before and after vaccine administration presumably to assess whether there was an appropriate increment in titer, during the study period. Similarly, only a small proportion of tests were repeated likely to assess the temporal trend in the titers. These findings suggests that these specialists are relying on the assessment of the baseline Td titers and polysaccharide vaccine responses (e.g., using the 23-valent unconjugated *Streptococcus pneumoniae* vaccine) for clinical decision making.

It is important to acknowledge that higher testing frequencies among certain specialties, such as immunology, nephrology, or oncology, may reflect the distinct immune status or clinical risk profiles of their patient populations. In such contexts, increased testing may be clinically justified rather than indicative of inappropriate overuse. Recognizing these population-specific factors is essential to accurately interpret observed specialty-based variations.

Outside of the assessment of patients with known or suspected immune compromising conditions, there are no strong indications for Td serological testing. The third highest patient group with Td serology utilization was patients on chronic renal replacement therapy (hemodialysis or peritoneal dialysis). The role of routine Td serology in this patient group is not established with no major guideline recommendations [20]. A German study in hemodialysis patients showed that this patient population has a poorer response to Td-containing vaccines compared to the general population with seroconversion rates ranging from 24–38% [21]. In a follow up study of hemodialysis patients, they found that 10 of 31 (32%) maintained protective diphtheria antibody levels (≥0.1 IU/mL), and 15 of 21 (71%) maintained protective tetanus antibody levels at 5 years post-vaccination, indicating a more rapid decline in specific antibody production compared to the general population [22]. This is consistent with our results, which show that Td titer results from tests requested by nephrologists are lower than those requested by most other providers.

At present, however, national guidance from major public health organizations continues to recommend the same revaccination protocol as the general population with Td-containing vaccine boosters every 8–10 years [23,24]. Further studies on the utility of Td titers, including whether they are truly representative of correlates of protection in this patient group and whether more frequent immunization boosters would be beneficial, are needed.

In contrast, the notably high geometric mean titers observed among hematology and pharmacy specialties may reflect sampling bias or targeted testing in select patient populations, such as those undergoing immune monitoring or receiving specialty care and may not be representative of the general testing population.

Most tests were requested by general practice physicians, more than a third of which were for patients who had documented Td vaccination within the past 10 years. Given the observed testing volumes, we suspect that most of these requests were unnecessary, likely reflecting limited awareness that titer measurements are not routinely indicated, particularly when vaccine records are available. This underscores the need for better vaccine recordkeeping and increased awareness of evidence-based testing guidelines among ordering providers. A scoping review study demonstrated that implementing diagnostic stewardship interventions such as electronic ordering restrictions and clinician education led to a significant reduction in unnecessary diagnostic tests, without compromising patient outcomes [25]. Our results suggest that such system level interventions could meaningfully reduce inappropriate serological testing in Alberta. In particular, integrating vaccination records into electronic medical systems, introducing automated prompts for test appropriateness, and providing clinician feedback through audit reports could serve as feasible, locally applicable strategies to enhance diagnostic stewardship.

Consistent with earlier epidemiological studies [12,26,27], our findings indicate that tetanus and diphtheria titers tend to be lower in older adults, though most maintained protective titers. A longitudinal study conducted by Amanna et al. (2007) followed individuals for up to 26 years in 45 subjects and demonstrated that antibody responses to tetanus and diphtheria decline gradually, with estimated half-lives of 11 (95% CI, 10–14) and 19 years (95% CI, 14–33), respectively [28]. This pattern may reflect a cohort effect, as individuals born in the 1920s and 1930s likely missed routine childhood immunization programs that were implemented later. Alternatively, the reduced antibody levels in older populations may be attributed to age-related immune senescence, where the capacity to sustain long-term antibody responses diminishes with advancing age [29]. While our findings regarding age-related decline in protective titers are consistent with existing literature, caution is warranted when interpreting seroprevalence as a direct correlate of immunity, as antibody levels may not fully reflect functional immune protection.

A large cross-sectional analysis of 546 adults also demonstrated that protective antibody levels against tetanus and diphtheria persist well beyond the currently recommended 10-year booster interval. In this study, approximately 97% of participants maintained partially protective titers (≥0.01 IU/mL), with mean levels of 3.6 IU/mL for tetanus and 0.35 IU/mL for diphtheria [30]. Notably, estimated half-lives were 14 years (95% CI, 11–17 years) for tetanus and 27 years (95% CI, 18–51 years) for diphtheria, and mathematical modeling predicted that 95% of the population would remain protected for at least 30 years post-vaccination. Our findings similarly showed that 88% with Td immunization >10 years ago had at least partially protective titers (≥0.01 IU/mL) and indicate that the standard 10-year adult booster schedule may be overly conservative and warrant re-evaluation.

Our study supports the development of targeted policy interventions such as EMR-based alerts, test-ordering restrictions, and clinician education modules may help improve the appropriateness of Td antibody testing. However, any changes to booster interval recommendations should be approached with caution and supported by prospective studies evaluating long-term immunity and clinical outcomes.

This study has several limitations. First, vaccination history may be incomplete or under-recorded in the dataset, potentially leading to misclassification of recent vaccine status. Notably, nearly 45% of patients lacked immunization records, which may have resulted in an overestimation of potentially unnecessary testing. Conversely, if missing records reflect vaccinations received outside the accessible databases, our estimates of over-testing could be conservative. As record linkage across systems was not feasible, no formal sensitivity analysis could be performed; however, we acknowledge that this uncertainty may affect the precision of our estimates. In addition, only a minority of patients had documented recent vaccination (e.g., 38.4% in general practice for diphtheria), which may limit the representativeness of this subgroup and warrants caution when generalizing findings related to vaccine-induced protection. Second, the small number of individuals tested before and after vaccination and lack of standardization of the interval between vaccination and repeat testing limits the generalizability of fold-change findings in antibody titers. Third, appropriateness and indication of Td serology requests was inferred by physician specialty and testing volumes rather than direct chart review and may not reflect the broader clinical context or rationale for testing. Finally, the data are limited to a single jurisdiction with a single government payer for serological testing and may not be generalizable to other healthcare systems with different testing practices, immunization policies, or ease of access to testing.

Future research should include prospective studies and randomized controlled trials to more robustly evaluate the clinical utility and cost-effectiveness of routine Td antibody testing and booster scheduling, particularly in immunocompromised populations where guidance remains limited.

## Conclusions

Our findings highlight that a considerable proportion of Td tests are ordered for individuals who were recently vaccinated despite guideline recommendations discouraging routine titer testing in low-risk, fully immunized patients. This trend underscores the need for greater adherence to diagnostic stewardship principles and enhanced awareness of long-lasting vaccine-induced immunity. In light of emerging evidence suggesting durable antibody responses persisting well beyond the current 10-year booster interval, future policy discussions should re-evaluate the frequency of routine booster dosing and antibody testing in adult populations. Further studies are warranted to optimize testing practices in hemodialysis patients and ensure alignment with both clinical need and immunological evidence.

## Supporting information

S1 Table Interpretation of titers for each assay (taken from the product insert)*. (DOCX)

S2 Table Number of tests ordered by each physician specialty. (DOCX)

S1 Fig Pre- and post-vaccination geometric mean titers for Td titers. (TIF)
