# Supplementary material for: Utilization of tetanus and diphtheria serology tests in Alberta, Canada: Patterns and implications
Source: PLoS One. 2025 Nov 21;20(11):e0336690. doi: 10.1371/journal.pone.0336690 (PMC12637915; doi:10.1371/journal.pone.0336690)
Supplement: S1 Table — (DOCX) [file pone.0336690.s001.docx]

**Table S1.** Interpretation of titers for each assay (taken from the product insert).*

| **Anti-Tetanus Toxoid ELISA (IgG)** | | **Anti-Diphtheria Toxoid ELISA (IgG)** | |
| --- | --- | --- | --- |
| <0.1 IU/mL | Insufficient immunity, booster advised. | <0.01 IU/mL | No protection. Individuals with no prior vaccination are advised to receive a primary series. |
| 0.1-0.5 IU/mL | Immunity given booster will provide long-term immunity. | 0.01-0.099 IU/mL | Uncertain protection. |
| >0.5-1.1 IU/mL | Sufficient immunity, booster vaccination advised in 2-5 years. | ≥ 0.1 IU/mL | Immunisation protection present. |
| >1.1-5.0 IU/mL | Sufficient immunity, booster vaccination advised in 5-10 years. | >1.0 IU/mL | Lon-term immunisation protection. |
| >5.0 IU/mL | Sufficient immunity, booster vaccination advised in 10 approximately 10 years. |  |  |

*For both kits, it is advised that alongside serologic findings, the vaccination history of the patient must always be taken into account (excerpt taken from the product insert).
