# Supplementary material for: Utilization of tetanus and diphtheria serology tests in Alberta, Canada: Patterns and implications
Source: PLoS One. 2025 Nov 21;20(11):e0336690. doi: 10.1371/journal.pone.0336690 (PMC12637915; doi:10.1371/journal.pone.0336690)
Supplement: S2 Table — (DOCX) [file pone.0336690.s002.docx]

**Table S2**. Number of tests ordered by each physician specialty.

| **Practitioner field** | **Test** | **Test requests**^☨^ | **%** |
| --- | --- | --- | --- |
| **General Practice** | Tetanus antitoxin | 1235 | 52.6 |
|  | Diphtheria antitoxin | 1163 | 55.6 |
| **Immunology** | Tetanus antitoxin | 548 | 23.3 |
|  | Diphtheria antitoxin | 480 | 22.9 |
| **Nephrology** | Tetanus antitoxin | 271 | 11.5 |
|  | Diphtheria antitoxin | 251 | 12.0 |
| **Pediatrics** | Tetanus antitoxin | 138 | 5.9 |
|  | Diphtheria antitoxin | 121 | 5.8 |
| **Hematology** | Tetanus antitoxin | 69 | 2.9 |
|  | Diphtheria antitoxin | 5 | 0.2 |
| **Pharmacy** | Tetanus antitoxin | 33 | 1.4 |
|  | Diphtheria antitoxin | 49 | 2.3 |
| **Respirology** | Tetanus antitoxin | 34 | 1.4 |
|  | Diphtheria antitoxin | 8 | 0.4 |
| **Infectious Diseases** | Tetanus antitoxin | 9 | 0.4 |
|  | Diphtheria antitoxin | 6 | 0.3 |
| **Internal Medicine** | Tetanus antitoxin | 2 | 0.1 |
|  | Diphtheria antitoxin | 3 | 0.1 |
| **Travel Medicine** | Tetanus antitoxin | 3 | 0.1 |
|  | Diphtheria antitoxin | 3 | 0.1 |
| **Critical Care** | Tetanus antitoxin | 2 | 0.1 |
| **Cardiac Surgery** | Tetanus antitoxin | 1 | 0.05 |
|  | Diphtheria antitoxin | 1 | 0.04 |
| **Emergency Medicine** | Diphtheria antitoxin | 1 | 0.05 |
| **ENT** | Tetanus antitoxin | 1 | 0.04 |
| **General Surgery** | Tetanus antitoxin | 1 | 0.04 |
| **Medical Genetics** | Tetanus antitoxin | 1 | 0.05 |
|  | Diphtheria antitoxin | 1 | 0.04 |
| **Obstetrics** | Tetanus antitoxin | 1 | 0.05 |
|  | Diphtheria antitoxin | 1 | 0.04 |

^☨^Some tests were obtained in the same patients more than once during the study period and these are recorded as separate test requests
