# Supplementary figures and images for: Utilization of tetanus and diphtheria serology tests in Alberta, Canada: Patterns and implications
Source: PLoS One. 2025 Nov 21;20(11):e0336690. doi: 10.1371/journal.pone.0336690 (PMC12637915; doi:10.1371/journal.pone.0336690)

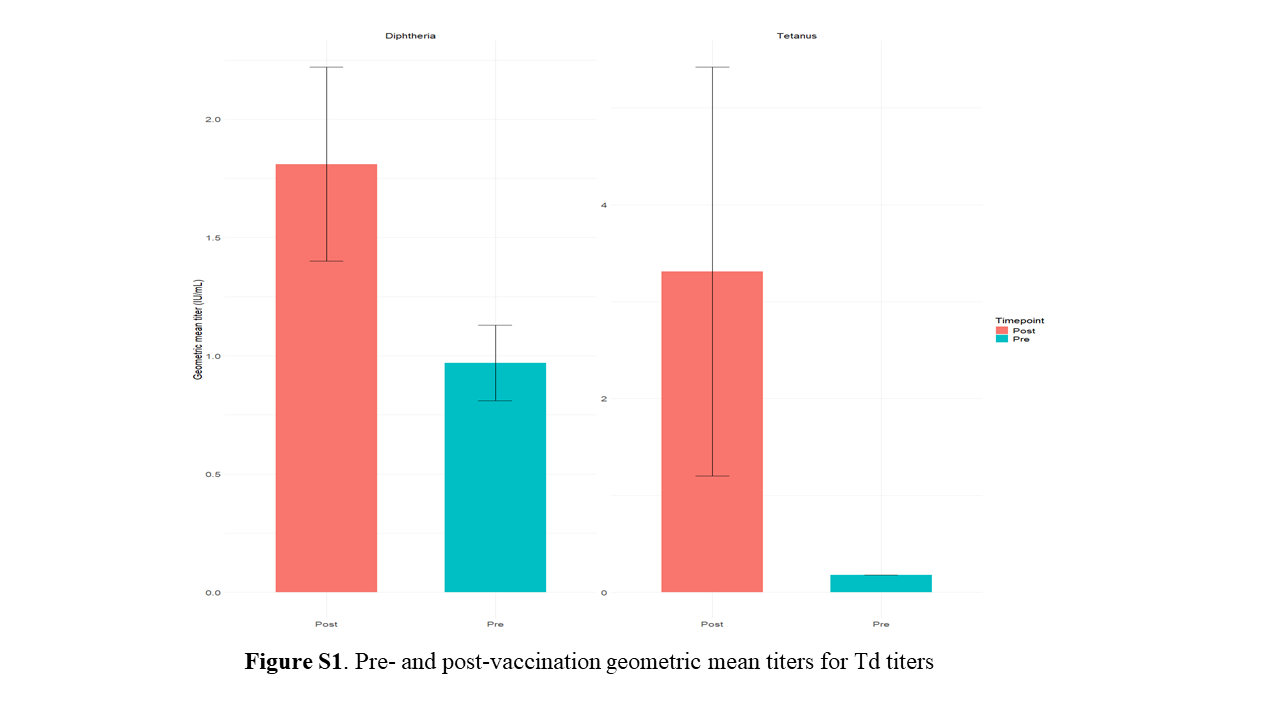

Supplement: S1 Fig — (TIF) [file pone.0336690.s003.TIF]
